# Academic stress and academic burnout in adolescents: a moderated mediating model

**DOI:** 10.3389/fpsyg.2023.1133706

**Published:** 2023-06-05

**Authors:** Xinhang Gao

**Affiliations:** School of Psychology, Shandong Normal University, Jinan, China

**Keywords:** academic stress, academic anxiety, academic self-efficacy, adolescence, academic burnout

## Abstract

**Objective:**

This study aims to investigate the relationship and mechanisms of action among academic stress, academic anxiety, academic self-efficacy, and academic burnout among adolescents.

**Methods:**

A study of 929 Chinese adolescents (53.71% males, mean age = 11.94 years, SD = 0.77) was conducted using the Study Stress Questionnaire, Academic Anxiety Subscale, Junior Middle School Students’ Learning Weariness Scale, and the Academic Self-efficacy Questionnaire.

**Results:**

①Academic stress was significantly and positively correlated with academic anxiety and academic burnout, and significantly and negatively correlated with academic self-efficacy. ②Academic anxiety partially mediated the relationship between academic stress and academic burnout. ③Academic self-efficacy significantly moderated the direct effect of academic stress on academic burnout, and higher academic self-efficacy could buffer the negative effect of academic stress. ④Academic self-efficacy significantly moderated the second half of the mediated model’s path (academic anxiety → academic burnout), that is, low academic self-efficacy amplified the risk effect of academic anxiety on academic burnout.

**Conclusion:**

Academic anxiety partially mediates the relationship between academic stress and academic burnout, and this mediating role is moderated by academic self-efficacy.

## Highlights

- Academic stress can positively predict academic burnout.
- This finding suggests that academic self-efficacy can reduce the association between academic stress and academic burnout and enhance the association between academic anxiety and academic burnout.
- Helping people gain a deeper understanding of the factors associated to academic burnout.
- Helping people to make more reasonable interventions to adolescents who have academic burnout.

## Introduction

Academic burnout is a persistent, negative, learning-related psychological state that occurs primarily in students (Zhang et al., 2007) and consists of three dimensions: emotional exhaustion, outside of study, and reduced personal achievement (Lin and Huang, 2014). In China, academic tiresome is a more colloquial expression for academic burnout, and the three manifestations of academic tiresome included in academic tiresome cognition, academic tiresome emotion, and academic tiresome behavior, which are three dimensions similar to academic burnout (Zhao, 2019). Also, academic burnout is measured by an instrument similar to academic tiresome (Yavuz and Dogan, 2014).

Chinese adolescents currently suffer from more severe academic burnout. Results of a survey conducted by the China Care for the Next Generation Working Committee in 2020 showed that more than 30% of elementary school students were bored with school and more than 70% of adolescent students have academic burnout, and many students experienced severe burnout or even refused to go to school due to the home quarantine during the epidemic that interrupted the normal rhythm of school attendance for adolescents (Sun et al., 2021; Swords et al., 2021). Regarding the impact of academic burnout on adolescents, current research has confirmed that academic weariness can lead to problematic behaviors such as absenteeism and school dropout (Wang et al., 2019), which can severely affect the entire family (Shen et al., 2012). In addition, some variables closely related to academic burnout are influential factors of adolescent mental health problems (Cheraghian et al., 2016), such as academic burnout can predict adolescent depression (Salmela-Aro et al., 2009) and neuroticism (Murberg and Bru, 2007). Adolescents, especially junior high school students, as a group just entering adolescence, have significant and rapid development and transition in psychological functioning (Gallagher et al., 2014). Chinese adolescents are prone to low academic control (Jose and Weir, 2013), burnout (Jiang et al., 2021), and school refusal behavior (Boussand et al., 2021) due to the number of courses they take, the difficulty of the curriculum, the pressure to advance to higher education (Dotterer et al., 2009; Lin, 2013), and high parental expectations (Huang et al., 2018). Therefore, this study aims to investigate the causes and mechanisms of adolescents’ academic burnout and to provide theoretical and empirical support for solving the problem of academic burnout.

### Academic stress and academic burnout

Previous studies have shown that students’ own poor learning foundation, low self-evaluation, and lack of interest and initiative in learning can lead to academic tiresome (Li, 2009; Zheng, 2013). Academic stress, an important stressor for adolescents (Ye et al., 2019; Nagamitsu et al., 2020), may be one of the factors influencing academic burnout. Although moderate stress can improve task performance (Henderson et al., 2012; Lin et al., 2022) and productivity (Kumari, 2021) in humans or animals, studies have shown that excessive academic stress not only causes negative cognitive attitudes toward academics (Savarese et al., 2019), affects students’ academic students’ performance (Khan et al., 2013), and reduces students’ academic performance (Canup, 2016); behaviorally leading to bad habits such as academic procrastination (Niazov et al., 2022), cell phone addiction (Shen et al., 2021), and also emotionally inducing depression (Jiang et al., 2021), reduced mental health (Aloia and McTigue, 2019), and even suicidal tendencies (Okechukwu et al., 2022). Thus, academic stress can affect adolescents in cognitive, behavioral, and emotional terms. Jessor et al. (2010) problem behavior theory states that problem behaviors (behavioral systems) can be directly influenced by individual internal personality factors (personality systems) as well as perceived external environmental factors (environmental perception systems). When an individual perceives academic stress, this perception can directly affect the adolescent’s behavioral system and produce burnout behaviors. The Transactional model of stress and coping (TSC) (Lazarus and Folkman, 1984) also argues that individuals cognitively assess stress after perceiving it. After feeling academic stress, adolescents may react negatively to this stress if they assess it as a threat (Walburg, 2014), which, in turn, may lead to academic burnout. In addition, there are also studies that show that academic stress is one of the factors that lead to academic burnout (Gonzálvez et al., 2018). Based on the above arguments, academic stress is one of the important factors that lead to students’ academic burnout. Therefore, this study will examine the relationship between academic stress and academic burnout in junior high school students and propose hypothesis H1: Academic stress in adolescents positively predicts academic burnout.

### Mediating effect of academic anxiety

Academic anxiety is a negative emotional state that students experience more frequently in academic situations (Gogol et al., 2017), encompasses anxiety about the school and learning environment and anxiety about academic activities (e.g., learning specific knowledge, exams, etc.) (Levine, 2008), and is a mediating variable between academic stress and negative academic performance (Nagpal and Sinha, 2016). On the one hand, as a typical negative academic emotion (Pekrun et al., 2002), academic anxiety can directly predict the occurrence of academic burnout (García-Fernández et al., 2011; Tao and Zhao, 2018; Pan and Zhang, 2021), and the higher the level of anxiety, the more individuals have manifestations of academic burnout, such as academic burnout (Fernández-Castillo, 2021), school refusal (Seçer and Ulaş, 2020), feelings of helplessness (Raufelder et al., 2018), and poor academic performance (Barbosa-Camacho et al., 2022). Processing efficiency theory states (Eysenck et al., 1987; Eysenck and Calvo, 1992) that highly anxious individuals are more likely to use negative learning strategies during learning, devoting limited cognitive resources to activities that are irrelevant to the learning task at hand, and choosing to allocate their attention to more irrelevant stimuli (Caviola et al., 2021). This is precisely in line with the behavioral manifestations of high academic burnout individuals, who appear to be mentally and behaviorally avoidant of the current learning task or learning process (Zhu et al., 2022). Therefore, we hypothesize that academic anxiety can influence academic burnout. On the other hand, anxiety as a physiological and psychological response triggered by stressors (Colich and McLaughlin, 2022), and stressful life events are a major stressor (Young and Dietrich, 2015). The higher the academic stress of adolescents, the higher their anxiety levels (Leung et al., 2010; Trevethan et al., 2022). Empirical studies have shown that adolescents increase academic stress (Park et al., 2012; Sun et al., 2012) and thus anxiety levels (Huberty, 2009) due to high homework loads, high expectations of teachers and parents, and lower academic performance. Therefore, we hypothesize that academic stress can influence academic anxiety. Regarding the emergence of this mechanism, the “context-process-outcome model” (Roeser et al., 1996) states that situational factors tend to influence individuals’ behavior by affecting their internal psychological processes. While stress is a hypothetical state in response to situational stimuli (Sarason, 1984), learning anxiety and academic burnout correspond to the psychological processes and outcomes of this model, respectively. We hypothesized that this mechanism of influence of academic stress would apply equally to academic burnout. For academic anxiety as a mediating variable, Fiorilli et al.'s (2020) study of school burnout in adolescents aged 13–17 showed that academic anxiety can mediate between trait emotional intelligence Trait emotional intelligence and school burnout. Dong and Liang et al. studied the causes of school burnout in junior high school students and found that academic stress was a mediating variable between anxiety and school burnout (Dong et al., 2021). In summary, academic anxiety may act as a mediating variable in the relationship between academic stress and academic burnout; therefore, this study proposes hypothesis H2: academic anxiety plays a mediating role in the relationship between academic stress and academic burnout.

### Moderating effect of academic self-efficacy

Academic self-efficacy (ASE) is the judgment and confidence in an individual’s ability to believe that he or she can successfully complete a specific academic task at a specific stage of learning (Schunk, 1991) and is the degree of belief in achieving the desired academic level (Weißenfels et al., 2022). Although academic anxiety affects the performance of academic burnout in middle school students, processing efficiency theory (Michael, 1982; Eysenck and Calvo, 1992) suggests that the relationship between anxiety levels and behavioral performance can be influenced by control or self-regulatory systems, and academic self-efficacy has the potential to act as a moderating variable for academic anxiety and academic burnout. Firstly, academic self-efficacy may play a moderating role between academic stress and academic burnout. First, according to Bandura’s self-efficacy theory (Bandura, 1977), individuals are able to be moderated by their self-efficacy when they face psychological and behavioral changes in response to stimuli, so individuals with high academic self-efficacy will have less burnout emotions and behaviors in response to academic stress. Second, studies on Chinese students have shown that self-efficacy can effectively regulate the relationship between stress and negative emotions such as depression, and stress and mental health (Schönfeld et al., 2019); studies on self-efficacy regulation of stress and adolescent life satisfaction showed that adolescents with high academic self-efficacy showed higher life satisfaction in the face of stress (Moksnes et al., 2019), whereas individuals with low self-efficacy showed higher life satisfaction in the face of stress. In addition, according to TSC (Lazarus and Folkman, 1984), academic self-efficacy can be used as a second evaluation mechanism for adolescents facing academic stress, and when academic self-efficacy is low, individuals are more likely to When academic self-efficacy is low, individuals are more likely to assess academic stress as a threatening factor and thus become academic burnout. Therefore, it can be hypothesized that academic self-efficacy can moderate the relationship between academic stress and academic burnout, and academic self-efficacy can buffer the reinforcing effect of academic stress on academic burnout.

**Figure 1:**
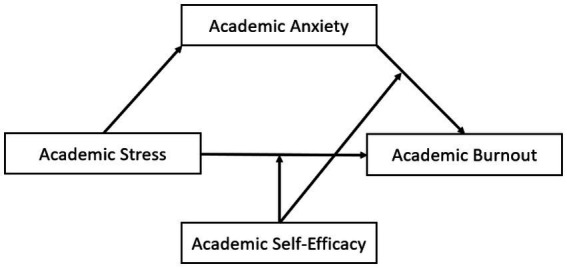
Moderated Mediating Model.

Furthermore, academic self-efficacy is considered to be a predictor and protective factor for adolescents’ internalizing and externalizing problems (Valle et al., 2006; Zee et al., 2017). Therefore, the relationship between academic anxiety as an internalized academic emotion (Lahdelma et al., 2021) and academic burnout may also be moderated by academic self-efficacy. First, research has shown that self-efficacy moderates the relationship between anxiety and academic performance (Barrows et al., 2013), and a decline in academic performance is one of the significant manifestations of academic burnout (Fu et al., 2002). Second, according to the control value theory of academic emotions (Pekrun, 2000; Pekrun et al., 2002), control cognition, which contains individuals’ expectations of the future (Roseman, 1996), is the main source of students’ academic emotions, and self-efficacy, as a control factor of cognition (Stenmark et al., 2021), can influence students’ academic emotions. Whereas in the academic life of adolescents, academic anxiety itself can affect academic performance and academic achievement (Hooda and Saini, 2017), individuals with low academic self-efficacy increase their assessment of threat (Putwain and Symes, 2012), further leading to academic burnout. Also according to the problem behavior theory (Jessor et al., 2010), the environmental perception system can interact with the personality system to produce problem behaviors, and academic self-efficacy, as a structure in the Personal Belief part of the personality system, can interact with academic anxiety, which can lead to academic burnout. Therefore, it can be hypothesized that academic self-efficacy can regulate the relationship between academic anxiety and academic burnout and buffer the reinforcing effect of academic anxiety on academic burnout. Based on the above analysis, this study proposes hypothesis H3: academic self-efficacy plays a moderating role between academic stress and academic burnout; specifically, the academic burnout of adolescents with high academic self-efficacy is more influenced by academic stress than those with low academic self-efficacy; this study proposes hypothesis H4: academic self-efficacy plays a moderating role between academic anxiety and academic burnout; specifically, the academic burnout of adolescents with high academic self-efficacy is more influenced by academic stress than those with low academic self-efficacy. Specifically, adolescents with high academic self-efficacy were more affected by academic anxiety than adolescents with low academic self-efficacy. Based on the problem behavior theory and transactional model, this study constructed a moderated mediation model (see Figure 1) based on the above assumptions as a way to explore the effect of learning stress on academic burnout and its mechanism of action.

## Materials and methods

### Design and procedure

We applied a survey design to achieve our research objectives. We used a whole-group sampling method and approached a junior high school in Shandong Province to collect data from their students, a sample of adolescents from this junior high school that was well represented. Participants were invited to provide their voluntary consent and then complete these measures. Participants did not provide any personal information that would allow researchers or organizations to identify them. Participants were led into the school’s microcomputer room by a schoolteacher and completed an electronic questionnaire on a computer after a trained master examiner read the instructions. All participation was voluntary, and each participant received a ballpoint pen as payment at the end of the test.

### Participants

Using the whole-group sampling method, all students in grades 6 and 7 of a junior high school in Shandong Province, a total of 982 adolescents, were selected to complete the questionnaire survey, and a total of 929 valid questionnaires were returned, with a valid return rate of 94.60%. Among them, 499 (53.71%) were male and 430 (46.29%) were female; 488 were in the first grade and 441 were in the second grade, aged 11 to 15 (11.94 ± 0.77). These adolescents came from urban, rural and rural areas, and it can be confirmed that the sample drawn is highly representative of the adolescent population.

### Measures

#### Academic stress

This study used the study stress questionnaire for middle school students (Xu et al., 2010) to measure adolescents’ academic stress. Twenty-one questions were included in the scale, including 4 dimensions of parents’ pressure, self-pressure, teacher pressure, and sociality pressure. The scale consists of 21 questions, including 4 dimensions: parents’ pressure, self-pressure, teacher pressure, and sociality pressure. Sample questions include parents pressure “My parents nag me a lot and want me to be an outstanding person,” self pressure “I think that going on to higher education is my only way out,” and teacher pressure “When I cannot answer the teacher’s questions, the teacher will criticize me. When I cannot answer the teacher’s questions, the teacher will criticize me,” sociality pressure “When I am worried, I feel that I do not have a friend to talk to.” The scale is scored on a 5-point scale, with 1 point for “Completely Not Conforming” and 5 points for “Completely Conforming.” The total Cronbach’s alpha coefficient of the scale was 0.95. We conduct confirmatory factor analysis (CFA) on 21 items; the average variance extracted (AVE) of each second-order factor is between 0.480 and 0.607, and the composite reliability (CR) is between 0.781 and 0.903. The results of the model showed that Chi-square/df = 6.233, *p* < 0.001, RMSEA = 0.075, SRMR = 0.042, GFI = 0.882, AGFI = 0.851, CFI = 0.922, TLI = 0.911, indicating that the results of CFA had good fitting indicators.

#### Academic anxiety

In this study, the academic anxiety subscale of the Mental Health Diagnostic Test (MHT), adapted by Zhou Bucheng (Zheng et al., 2004), was used to measure adolescents’ academic anxiety. The scale consists of 15 questions (e.g., “Do you always think about tomorrow’s homework when you go to bed at night?”). The scale is scored on a two-point scale, with “Yes” scoring one and “No” scoring zero, with higher scores indicating higher levels of anxiety. The Cronbach’s alpha coefficient for this scale was 0.86. We conduct confirmatory factor analysis (CFA) on 21 items; the average variance extracted (AVE) is 0.299, and the composite reliability (CR) is 0.852. The results of the model showed that Chi-square/df = 4.297, p < 0.001, RMSEA = 0.060, SRMR = 0.043, GFI = 0.946, AGFI = 0.926, CFI = 0.926, TLI = 0.11, indicating that the results of CFA had good fitting indicators.

#### Academic burnout

This study used Junior Middle School Students’ Learning Weariness Scale (Zhao, 2019) to measure adolescents’ academic tiresome. The questionnaire has 17 items including 3 dimensions of academic tiresome cognition, academic tiresome emotion, and academic tiresome behavior. Sample questions such as academic tiresome cognition “I do not get any pleasure from studying,” academic tiresome behavior “I often try to avoid studying,” and academic tiresome emotion “Studying often makes me feel physically and mentally exhausted.” The scale was scored on a 5-point Likert scale, with all positive scores, one score for “Not at all” and five scores for “Fully,” and the higher the total score, the more serious the degree of academic burnout. The total Cronbach’s alpha coefficient of the scale was 0.95. We conduct confirmatory factor analysis (CFA) on 21 items; the average variance extracted (AVE) of each second-order factor is between 0.571 and 0.670, and the composite reliability (CR) is between 0.890 and 0.903, indicating that the aggregation validity is high. The results of the model showed that Chi-square/df = 7.114, *p* < 0.001, RMSEA = 0.081, SRMR = 0.046, GFI = 0.906, AGFI = 0.874, CFI = 0.938, TLI = 0.927, indicating that the results of CFA had good fitting indicators.

#### Academic self-efficacy

The academic self-efficacy questionnaire, developed by Pintrich and De Groot (1990) and revised in Chinese by Liang (2000), is a 22-item scale containing two dimensions: self-efficacy of academic ability and self-efficacy of academic behavior. Sample questions such as “I believe I have the ability to do well in my studies” and “I always like to check whether I have mastered what I have learned through self-questioning when studying” are scored on a 5-point scale. The higher the total score, the stronger the academic self-efficacy. The total Cronbach’s alpha coefficient of the scale was 0.94. We conduct confirmatory factor analysis (CFA) on 21 items; the average variance extracted (AVE) of each second-order factor is between 0.448 and 0.634, and the composite reliability (CR) is between 0.853 and 0.950. The results of the model showed that Chi-square/df = 7.212, *p* < 0.001, RMSEA = 0.082, SRMR = 0.064, GFI = 0.865, AGFI = 0.834, CFI = 0.922, TLI = 0.912, indicating that the results of CFA had good fitting indicators.

### Data analysis

First, the common method deviation test was performed using SPSS 22.0, and descriptive statistics and correlation analysis were performed for the main variables. The common method bias test calculated according to Harman’s one-way test showed that there were 10 factors with eigenvalues greater than 1. The first principal component explained 29.89% of the total variance, which is below the critical value of 40%, so it can be concluded that there is no significant common method bias problem in this study. After that, Model 15 in SPSS macro program process v3.3 prepared by Hayes and Scharkow (2013) was used to perform the moderated mediation model test and Bootstrap method (2000 replicate samples with confidence interval set to 95%) was used to test the significance of the mediation effect.

## Results

### Descriptive and correlation analyses

As shown in Table 1, the results of descriptive statistics and correlation analysis showed that there was a significant positive correlation between academic stress, academic anxiety, and academic burnout, and a significant negative correlation between academic self-efficacy and academic stress, academic anxiety, and academic burnout.

**Table 1:** Average, standard deviation, and correlation coefficient of each variable (*N* = 929).

	*M*	*SD*	1	2	3	4
1. Academic stress	2.56	0.94	–			
2. Academic anxiety	1.63	0.26	0.51^***^	–		
3. Academic burnout	1.85	0.71	0.40^***^	0.30^***^	–	
4. Academic self-efficacy	3.63	0.73	−0.39^***^	−0.26^***^	−0.67^***^	–

^*^p < 0.05, ^**^p < 0.01, and ^***^p < 0.001.

### Mediation effect test

In the Process macro proposed by Hayes and Scharkow (2013), the mediating effect of learning anxiety was tested using Model 4. The Bootstrap test (a statistical method for multiple repetition sampling) was chosen and set with repetitions of 2000 and 95% confidence intervals. The results showed that the predictive effect of academic stress on academic burnout was significant (*β* = 0.38, *t* = 13.40, *p* < 0.001), and the predictive effect of academic stress on academic burnout remained significant when the mediating variable academic anxiety was introduced (*β* = 0.32, *t* = 9.74, *p* < 0.001), academic stress had a significant predictive effect on academic anxiety (*β* = 0.50, *t* = 17.98, *p* < 0.001), and academic anxiety had a significant positive predictive effect on academic burnout (*β* = 0.12, *t* = 3.70, *p* < 0.001). The upper and lower limits of Bootstrap 95% confidence intervals for the direct effect of academic stress on academic burnout and the mediating effect of academic anxiety did not contain 0 (see Table 2), indicating that academic stress not only directly predicted academic burnout Academic anxiety partially mediates between academic stress and academic burnout, with a mediating effect value of 0.06 and 95% confidence interval of (0.03, 0.10), indicating that the mediating effect of academic anxiety was significant, accounting for 15.79% of the total effect and 18.75% of the direct effect value.

**Table 2:** Total, direct and indirect effects.

	Effect	Boot SE	Boot LLCI	Boot ULCI
Total effect	0.38	0.03	0.00	0.32
Direct effect	0.32	0.03	0.00	0.25
Indirect effect	0.06	0.02	0.03	0.10

### Academic stress and academic burnout: testing for moderated-mediation

Model 15 in the process macro program prepared by Hayes was used to test for a moderating mediating effect. The results showed that equation 1 was significant overall (*F* (1, 927) = 323.24, *p* < 0.001) and learning stress was a significant positive predictor of learning anxiety (β = 0.50, *t* = 17.98, *p* < 0.001). Equation 2 was significant overall (*F* (5, 923) = 169.84, *p* < 0.001), with learning stress significantly and positively predicting academic burnout (*β* = 0.13, *t* = 4.86, *p* < 0.001) and learning anxiety significantly and positively predicting academic burnout (*β* = 0.07, *t* = 2.83, *p* < 0.01), thus learning anxiety mediated the relationship between learning stress and academic burnout. The interaction term between academic stress and academic self-efficacy was a significant negative predictor of academic burnout (*β* = −0.08, *t* = −3.05, *p* < 0.01), and the interaction term between academic anxiety and academic self-efficacy was a significant positive predictor of academic burnout (*β* = 0.05, *t* = 2.05, *p* < 0.05), indicating a significant moderating effect of academic self-efficacy. The results are shown in Table 3.

**Table 3:** The moderated-mediating effect of academic stress on academic burnout.

Predictor variable	Equation 1 academic anxiety (M)			Equation 1 academic burnout(Y)		
	*β*	*t*	95% CI	*β*	*t*	95% CI
Academic stress	0.50	17.98^***^	[0.45, 0.56]	0.13	4.86^***^	[0.08, 0.19]
Academic self-efficacy				−0.55	−22.49^***^	[−0.60, −0.51]
Academic stress ^*^ academic self-efficacy				−0.08	−3.05^**^	[−0.13, −0.03]
Academic anxiety				0.07	2.83^**^	[0.02, 0.13]
Academic anxiety ^*^ academic self-efficacy				0.05	2.05^*^	[0.00, 0.10]
*R* ^2^	0.26			0.48		
*F*	323.24^***^			169.84^***^		

^*^p < 0.05, ^**^p < 0.01, and ^***^p < 0.001.

**Figure 2:**
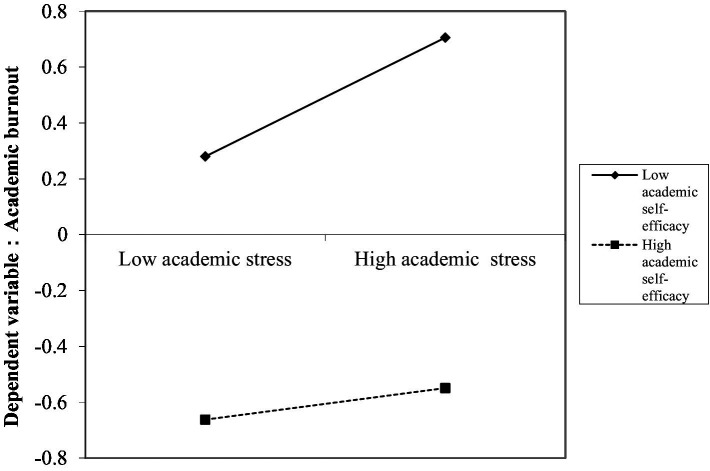
Model of the test for simple slopes showing the moderating influence of academic self-efficacy of the association between academic stress and academic burnout.

After standardizing the study variables, the study divided the subjects into low (*Z* ≦ -1*SD*) and high (*Z* ≧ 1*SD*) subgroups according to the standardized scores controlling for academic self-efficacy for simple slope analysis. First, we explored how academic self-efficacy moderated the direct effect of academic stress on academic burnout. The results found (see Figure 2) that the predictive effect of academic stress on academic burnout was significant for adolescents when the level of academic self-efficacy was low (*simple slope* = 0.23, *SE* = 0.04, *p* < 0.001) and insignificant for adolescents when the level of academic self-efficacy was high (*simple slope* = 0.06, *SE* = 0.04, *p* = 0.10). This indicates that the lower the academic self-efficacy of middle school students, the greater the effect of academic stress on academic burnout, and conversely, at high levels of academic self-efficacy, the effect of academic stress on academic burnout was not significant.

**Figure 3:**
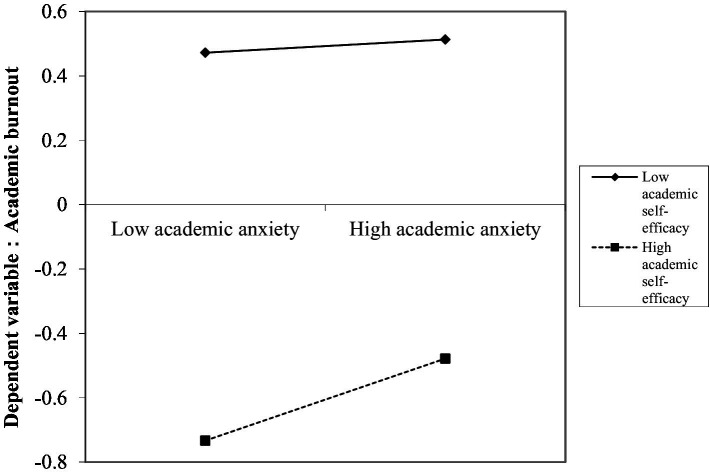
Model of the test for simple slopes showing the moderating influence of academic self-efficacy of the association between academic anxiety and academic burnout.

The study further went on to analyze the moderating effect of academic self-efficacy on the relationship between academic anxiety and academic burnout in the second half of the mediated model path (see Figure 3). It was found that for middle school students with low academic self-efficacy levels, academic anxiety was not a significant predictor of academic burnout (*simple slope* = 0.02, *SE* = 0.04, *p* = 0.58); for adolescents with high academic self-efficacy, academic anxiety was a significant positive predictor of academic burnout (*simple slope* = 0.13, *SE* = 0.04, *p* = 0.0004). This suggests that the higher the academic self-efficacy of middle school students, the greater the effect of learning anxiety on academic burnout may be, and on the contrary, at low levels of academic self-efficacy, the effect of learning anxiety on academic burnout may not have a significant effect.

The bias-corrected bootstrap confirmed that the indirect relationship between academic stress and academic burnout through anxiety was moderated by academic self-efficacy. To be specific, with the improvement of self-efficacy, the indirect effect was stronger (low level of academic self-efficacy: *β* = 0.011, SE = 0.027, 95% CI = −0.040 to 0.065; medium level of academic self-efficacy: *β* = 0.038, SE = 0.015, 95% CI = 0.010 to 0.067; high level of academic self-efficacy: *β* = 0.064, SE = 0.017, 95% CI = 0.032 to 0.100). The results support the hypotheses developed in this research.

## Discussion

This study constructed a moderated mediation model based on the problem behavior theory and transactional model, administered to adolescent students in grades 6 and 7, examined the relationship between academic stress and academic burnout, and tested the mediating role of academic anxiety and the moderating role of academic self-efficacy. The results of the study both expand the application of the model and contribute to the understanding of the critical issue of how academic stress affects academic burnout among junior high school students, and provide important insights into how to intervene in adolescents’ academic burnout.

### The relation between academic stress and academic burnout

Adolescents experience a variety of stressors, and academic stress is one of the most significant sources of stress (Ang and Huan, 2006). The present study found that academic stress in adolescents can significantly influence academic burnout, and the findings support hypothesis H1, which is consistent with previous findings (Kim and Jang, 2016; Gonzálvez et al., 2018; Jiang et al., 2021). In terms of the three components of academic burnout, this result also supports the problem behavior theory (Jessor, 1987) and the transactional model (Lazarus and Folkman, 1984). On the one hand, in terms of the behavioral component of aversion, the problem behavior theory suggests that the stress perception system directly influences the occurrence of problem behaviors and that the perception of stress also directly influences academic burnout behaviors when adolescents are in a stressful learning environment. On the other hand, in terms of the emotional and cognitive components of academic burnout, the transactional model (Lazarus and Folkman, 1984) suggests that individuals may assess learning stress as a threatening factor (Tadeo-Álvarez et al., 2019), resulting in academic burnout emotions and academic burnout cognitions (Hodge-Windover, 2018). In summary, academic stress has a direct impact on the cognitive, emotional, and behavioral aspects of academic burnout, which can increase the level of adolescents’ academic burnout.

### The mediating role of academic anxiety

The present study also revealed the mediating role of academic anxiety between academic stress and academic burnout, that is, academic stress can influence academic burnout not only directly and directly but also indirectly through academic anxiety, and the findings support hypothesis H2. First, the present study found that academic stress can positively predict academic anxiety. The higher the academic stress of adolescents, the higher their anxiety levels will be, which is consistent with previous research findings (Khng, 2017; Dube et al., 2018; Pan and Zhang, 2021). According to the transactional model, learning anxiety is also an emotional response that individuals make after assessing stress (Stetler and Guinn, 2020). When the assessment of stress or the ability to regulate emotions is abnormal, individuals are prone to anxiety (Bhat, 2017). Second, the present study also found that academic anxiety positively predicted academic burnout, consistent with previous research (Fernández-Castillo, 2021). This is because negative academic emotions trigger more in negative actual performance (e.g., academic burnout), and a decrease in the level of academic anxiety in individuals will help them to stay motivated and active in their studies, contributing to a reduction in academic burnout (Steel, 2010). It is evident that learning anxiety acts as a bridge between learning stress and academic burnout; the more learning stress adolescents are subjected to, the more likely they are to develop learning anxiety, and this anxiety subsequently leads to higher levels of academic burnout. This result supports the stress process model (Pearlin et al., 1981). This model suggests that stress can affect individuals both directly and indirectly by increasing certain negative psychological resources such as anxiety (Aneshensel and Avison, 2015). Here, academic anxiety, as a result of the perception of a stressful environment (Haikalis et al., 2022), is able to mediate the process of academic stress and academic burnout. In summary, academic stress can indirectly influence academic burnout through the mediating role of academic anxiety.

### The moderating role of academic self-efficacy

The present study also found that academic self-efficacy moderated both the “academic stress and academic burnout” and “academic anxiety and academic burnout” pathways. First, academic self-efficacy buffered the negative effects of academic stress on academic burnout, that is, as academic self-efficacy increased, the predictive effect of academic stress on academic burnout decreased. Previous research has shown that Problem solving can increase with stress from assignments and workload for individuals with low self-efficacy, whereas this association is not significant for individuals with higher self-efficacy (Zhao et al., 2015), so the academic burnout of adolescents with low academic self-efficacy increases with academic stress The association was not significant for adolescents with high academic self-efficacy (Zhao et al., 2015). Research has shown that levels of academic burnout are strongly related to life satisfaction and problem solving (Lian et al., 2014; Xiaoman et al., 2021), and longitudinal studies of adolescents suggest that self-efficacy moderates the relationship between stress and life satisfaction, with life satisfaction significantly decreasing with increasing stress for individuals with low self-efficacy, whereas for individuals with high self-efficacy, life satisfaction is not significantly affected by stress (Burger and Samuel, 2017). The cognitive theory of stress proposed by Lazarus suggests that individuals with high self-efficacy perceive stressful events as challenges rather than threats and respond with positive behaviors or psychological states (Lazarus and Folkman, 1984; Homburg and Stolberg, 2006; Peng et al., 2015). For the present study, individuals with high academic self-efficacy perceive academically stressful events as challenges rather than threats, and reduce levels of academic burnout and respond to academic stress with positive psychological and academic performance. Therefore, academic self-efficacy can act as a moderating variable to regulate the relationship between academic stress and academic burnout.

In the relationship between academic anxiety and academic burnout, academic self-efficacy, rather than buffering the effect of academic anxiety on academic burnout, enhanced the association, a result inconsistent with the expectation of Hypothesis 4, that is, the positive predictive relationship between academic burnout and academic anxiety became more pronounced as self-efficacy increased. The results are also inconsistent with some previous research, where a study of children and adolescents showed that math self-efficacy buffered the negative effects of anxiety on academic performance, with anxiety negatively predicting math test scores among individuals with low self-efficacy levels only, whereas for individuals with high self-efficacy, anxiety did not predict lower test scores (Galla and Wood, 2012; Pérez Fuentes et al., 2020). However, there are studies that support this result, as Burns et al.'s (2021) study noted that science self-efficiency negatively moderated science anxiety and science achievement, and for students with high science self-efficiency, high anxiety for students with high science self-efficiency, science achievement was significantly lower than that of students with low anxiety, whereas for students with low science self-efficiency, science achievement was significantly higher for students with high anxiety than for students with low anxiety, with anxiety having a potentially motivating effect on students with low self-efficacy (Burns et al., 2021). A study by Barrows et al. (2013) also found that self-efficacy did not mitigate the effects of test anxiety on test scores.

The enhancement effect produced by academic self-efficacy in this study can be explained in two ways: on the one hand, the Reverse risk-buffering model (RBSM) suggests that the protective effect of protective factors is undermined when risk factors are too high (Vanderbilt-Adrian and Shaw, 2008), and learning anxiety, as a risk factor, may undermine the protective effect of academic self-efficacy on predictive and protective effects of adolescents’ internalizing problems (Valle et al., 2006; Zee et al., 2017). On the other hand, Pekrun’s control-value theory of academic emotions suggests that self-efficacy is one of the sources of academic emotions (Pekrun, 1998; Pekrun, 2000) and can moderate the relationship between academic emotions and academic outcomes by changing expectations (Pekrun et al., 2002). And Pekrun et al. (2002) argued that excessive expectations can trigger anxiety in individuals when they mean facing possible failure. That is, individuals with high academic self-efficacy have high expectations for outcomes (Doménech-Betoret et al., 2017) and the anxiety they generate when faced with complex or difficult tasks may make them feel that expectations are difficult to meet and thus create anxiety, whereas individuals with low academic self-efficacy have high levels of anxiety themselves and have low expectations for outcomes, so anxiety does not lead to further academic burnout. Therefore, for middle school students with high academic self-efficacy, the effect of learning anxiety on academic burnout may be greater.

## Limitations and practical implications

In this study, a moderated mediation model was constructed to examine the mediating process of “academic stress → academic anxiety → academic burnout” and the moderating role of academic self-efficacy. The results showed that the mediating effect of academic anxiety between academic stress and academic burnout was significant, and the moderating effect of academic self-efficacy in the direct path and the second half of the mediating path was significant, which helps to understand the relationship between learning stress and academic burnout and its internal mechanisms. There are several limitations of this study that could be improved in further research. First, this study used a cross-sectional design and was unable to test the stability of this mechanism of action across time; future studies could be administered to these participants again after a certain period of time to explore the stability of this mechanism of action across time; second, all of the variables explored in this study were related to academics, and according to ecosystem theory (Guy-Evans, 2020), home environment, teacher instruction, peer relationships etc., may all have an impact on students’ academic burnout, so future research could explore the impact of these variables on academic burnout. Third, although research in the field of managerial psychology has shown that stress has a positive U-curve with performance (Jamal, 1984; AbuAlRub, 2004), studies of teachers have shown that stress negatively predicts teaching performance (Kumari, 2021). The present study found that academic stress positively predicted academic burnout, a linear relationship, so future research could explore whether there is a nonlinear relationship between academic stress and academic burnout. Finally, the use of self-report questionnaires to collect data may have left subjects subject to social approbability, thus not accurately obtaining their true data, which needs to be improved in future studies.

Nevertheless, this study has strong practical implications. Because of the current high levels of academic burnout among some adolescents, this study can provide targeted suggestions and insights for preventing and intervening in adolescents’ academic burnout. The results of the study show that, first, we can reduce the level of academic burnout by reducing the academic stress of adolescents, such as reducing extracurricular assignments. Second, we can equip adolescents with ways to regulate their emotions and reduce academic boredom by reducing academic anxiety. In addition, according to the results of the study, academic self-efficacy is effective in boosting confidence and coping with external stress effectively, but if students have high levels of academic anxiety, instantly students with high academic self-efficacy will increase the expression of academic burnout as their anxiety level increases. Therefore, for adolescents with high academic self-efficacy, although they can cope with external pressure effectively, they need to pay attention to the regulation of their academic anxiety, so it is more important to provide them with emotional psychological guidance to alleviate their academic anxiety levels; for adolescents with low academic self-efficacy, they need to stimulate their academic self-efficacy as well as pay attention to their own internal emotional regulation. In conclusion, the intervention and prevention of academic burnout in junior high school students should not focus on one aspect only, but should be carried out in an integrated and systematic way from three aspects: academic stress, academic anxiety, and academic self-efficacy.

## Data availability statement

The original contributions presented in the study are included in the article/Supplementary material, further inquiries can be directed to the corresponding author.

## Ethics statement

The studies involving human participants were reviewed and approved by the ethics committee at Shandong Normal University. Written informed consent to participate in this study was provided by the participants' legal guardian/next of kin. Written informed consent was obtained from the minor(s)' legal guardian/next of kin for the publication of any potentially identifiable images or data included in this article.

## Author contributions

XG: conceptualization, methodology, supervision, validation, resources, data curation, formal analysis, investigation, validation, and writing—original draft.

## Funding

This study was financially supported by National College Students’ Innovation and Entrepreneurship Training Program of China [grant number: 202210445011]. Shandong Normal University Undergraduate Research Fund Project in 2023 [grant number: BKJJ2022083].

## Conflict of interest

The author declares that the research was conducted in the absence of any commercial or financial relationships that could be construed as a potential conflict of interest.

## Publisher’s note

All claims expressed in this article are solely those of the authors and do not necessarily represent those of their affiliated organizations, or those of the publisher, the editors and the reviewers. Any product that may be evaluated in this article, or claim that may be made by its manufacturer, is not guaranteed or endorsed by the publisher.
